# Modelling the Evolution of COVID-19 in High-Incidence European Countries and Regions: Estimated Number of Infections and Impact of Past and Future Intervention Measures

**DOI:** 10.3390/jcm9061825

**Published:** 2020-06-11

**Authors:** Juan Fernández-Recio

**Affiliations:** Instituto de Ciencias de la Vid y del Vino (ICVV), CSIC-Universidad de La Rioja-Gobierno de La Rioja, Ctra. Burgos Km 6, 26007 Logroño, Spain; juan.fernandezrecio@icvv.es; Tel.: +34-941-894980

**Keywords:** COVID-19, virus, epidemiology, disease transmission model, intervention measures

## Abstract

A previously developed mechanistic model of COVID-19 transmission has been adapted and applied here to study the evolution of the disease and the effect of intervention measures in some European countries and territories where the disease has had a major impact. A clear impact of the major intervention measures on the reproduction number (*R*_t_) has been found in all studied countries and territories, as already suggested by the drop in the number of deaths over time. Interestingly, the impact of such major intervention measures seems to be the same in most of these countries. The model has also provided realistic estimates of the total number of infections, active cases and future outcomes. While the predictive capabilities of the model are much more uncertain before the peak of the outbreak, we could still reliably predict the evolution of the disease after a major intervention by assuming the subsequent reproduction number from the current study. A greater challenge is to foresee the long-term impact of softer intervention measures, but this model can estimate the outcome of different scenarios and help to plan changes for the implementation of control measures in a given country or region.

## 1. Introduction

The ongoing pandemic expansion of the new SARS-CoV-2 virus has caused over 3,500,000 detected cases of coronavirus disease 2019 (COVID-19) and claimed over 240,000 lives worldwide as of 5 May 2020 [1]. Since the first epidemic wave has dramatically hit a large number of countries, we can learn from all available epidemiological data to understand the evolution of the disease in order to better prepare the healthcare capacities for future pandemic waves. A recent model of SARS-CoV-2 transmission based on estimates of seasonality, immunity and cross-immunity from past virus pandemic data indicates the high risk of recurrent outbreaks in the coming years, according to different scenarios [2]. Before the availability of effective vaccines and pharmaceutical treatments, the transmission of the disease can only be controlled by prevention measures, like personal hygiene habits, social distancing, case detection and isolation, or by contact tracing. Strict social distancing enforced by the authorities has been the strategy of choice by the majority of countries in this first epidemic outbreak, implemented as different measures in each country (imposing or encouraging home confinement, closing schools and workplaces and banning assemblies and gatherings, among others). Given that this usually involves a strong economic and behavioral impact in a society, it is urgently necessary to understand the evolution of the disease in each country or territory and to estimate the effect of implementing such control measures. A major problem is that in most countries, only a small portion of the infected individuals is detected, and as a consequence, undetected contagious patients are the major cause of the expansion of the disease, which is the main limitation in foreseeing the effect of intervention measures. In the absence of large-scale detection tests able to be applied to an entire country population, mathematical modeling can help to estimate the dynamics of infections based on available epidemiological data. Indeed, a recent networked dynamic metapopulation model using Bayesian inference and based on reported infections and mobility data suggests a high proportion of undocumented infections in the first SARS-CoV-2 outbreak in China [3]. That model compared the initial transmission dynamics with those after greater control measures were introduced, showing a clear reduction in the transmission rate and the effective reproduction number. However, the specific impact of the different control measures was difficult to analyze at that time.

In this context, a Bayesian mechanistic model was recently proposed to estimate the effects of specific intervention measures on the reproduction number in 11 countries by inferring the number of infections from the observed deaths over time [4], assuming an infection fatality ratio (IFR) that was different for each country, adjusted to the age distribution of their population. A major assumption of that study was that the intervention measures had the same relative effect in all countries, which is not necessary true. For instance, according to large-scale mobile phone data [5], the effect of lockdown measures on the mobility of the population was clearly different in each country. The level of isolation of transmitting cases not only depends on the intervention to the general population, but also on the percentage of detected patients, which might make the isolation of such patients more or less effective.

Another limitation of the mentioned study [4] is that, at that moment, the pandemic was in the early stages of its evolution. Since the peak of the outbreak had not yet been reached in the studied countries, the impact of intervention could be followed based solely on small changes in the slope of the growth of reported deaths over time, with a large degree of uncertainty. Moreover, for some of the intervention measures, an insufficient time had passed to see a real impact on the number of deaths. As a consequence, the impact of the intervention measures of most of the countries were probably underestimated, and the forecast anticipated a larger number of expected infections and deaths than those found in the following weeks.

Here, a new study has been performed by using current data on the five European countries with the highest number of total cases (Spain, Italy, UK, France and Germany), in which the disease has significantly evolved and seems to have overpassed the outbreak peak. In this work, the analysis has also included Iceland and the Spanish region of La Rioja, with comparable populations, in which the number of reported cases in proportion to their population is among the highest ones in Europe. The model has been adapted by fitting it individually to each country, and by simplifying the number of intervention measures to evaluate. The result is a model that nicely explains the evolution of the disease and that can describe the effective impact of the intervention measures and estimate the outcome of future interventions.

## 2. Methods

### 2.1. Data Collection

Daily real-time death data was gathered from the ECDC (European Centre of Disease Control). Table 1 shows the up-to-date (5 May 2020) detected cases and reported deaths for high-incidence countries (Spain, Italy, United Kingdom, Germany, France, Iceland), as well as for La Rioja, the region in Spain with the highest number of incidences relative to its population. In the latter, the model is also applied to the data after excluding the population from elderly retirement homes with reported cases. The high incidence of cases in elderly retirement homes in European countries suggests that transmission of the disease has followed different patterns in these cases, and the impact of general lockdown measures might not be the same as in the general population. Since this data is available for this work only in the case of La Rioja, it has been considered worthy of inclusion in the analysis.

### 2.2. Estimating New Infections and Deaths over Time

The major details of the model have been described elsewhere [4]. Basically, the expected number of deaths *D_i_* in a given day *i* is a function of the number of infections *I_j_* occurring in the previous *j =* 1…*i*−1 days, according to a previously calculated infection-to-death (*ITD*) probability distribution and an estimated infection fatality ratio (IFR) for each country (Equation (1)).

(1) $$D_{i}=\overset{i-1}{\underset{j=1}{\sum }}I_{j}\cdot IFR\cdot ITD_{i-j}$$

**Figure 1:**
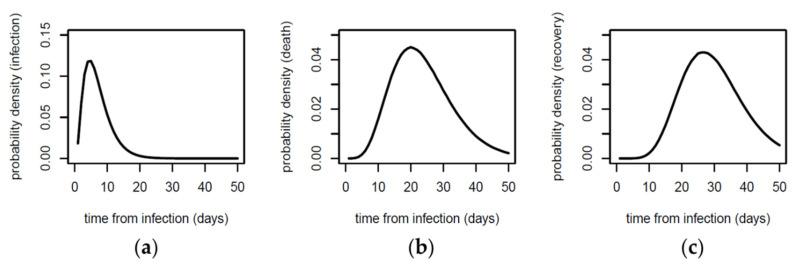
Probability distributions used in this work for (**a**) serial interval, (**b**) infection-to-death and (**c**) infection-to-recovery.

The infection-to-death (*ITD*) probability distribution was modelled, using previous epidemiological data from the early outbreak in China [6,7], by adding up two independent distributions: (i) the infection-to-onset (incubation period) distribution, estimated as a Gamma probability density function with mean 5.1 days and a coefficient of variation 0.86; and (ii) the onset-to-death (time between onset and death) distribution, estimated as a Gamma probability density function with a mean of 18.8 days and a coefficient of variation 0.45 [4]. The resulting *ITD* probability distribution is shown in Figure 1b.

The infection fatality ratio (IFR) is the probability of death for an infected case. The values used here for COVID-19 are derived from previous estimates [8] and are calculated for each country according to the age-distribution population [6,7]. For La Rioja, the same IFR is used as for Spain. For Iceland, the general IFR value of 0.657% was initially used, as previously reported by combining estimates of case fatality ratios with information on infection prevalence in China [7]. This IFR estimate provided an expected number of infected and active cases close to the detected ones. However, the case fatality rate (CFR), an empirical value obtained from the number of deaths over the detected cases, gives a value of CFR = 0.556% for Iceland, and the true IFR could be even smaller [9], so this CFR value (0.556%) was finally used as a better estimate of IFR in Iceland. The IFR values used here for the different countries are shown in Table 2.

In this model, the expected number of new infections *I_j_* occurring in a given day *j* is a function of the number of infections *I_k_* in the previous *k =* 1…*j*−1 days, according to a serial interval (*SI*) distribution, and the reproduction number (*R_t_*) (Equation (2)).

(2) $$I_{j}=\overset{j-1}{\underset{k=1}{\sum }}I_{k}\cdot R_{t}\cdot SI_{j-k}$$

The *SI* probability distribution was modelled as a Gamma probability density function with a mean of 6.5 days and a coefficient of variation 0.62 (Equation (2)), as previously described [4], and is shown in Figure 1a. The reproduction number (*R_t_*) had an initial constant value *R_0_* (a parameter that was left to optimize during the fitting of the model independently for each country), and was assumed to change after an intervention to another value which was kept constant over time until a new intervention (the new *R_t_* value after a given intervention is also a parameter to optimize during the model fitting). For simplicity, the entire population was assumed to be susceptible to infection during model fitting, but for long-term predictions based on the resulting model parameters (see Section 3.5), the infected cases were assumed to be protected from further infection.

### 2.3. Model Fitting

The expected number of deaths obtained from the above described model (Equations (1) and (2)) were fitted to the observed number of daily deaths, and were assumed to follow a negative binomial distribution, as previously described [4]. In the case of Iceland, both the observed number of daily deaths and the cumulative number of observed deaths were fitted, because the observed daily deaths alone did not provide reliable fitting due to the small size of the sample. Following the initial procedure [4], the model included only observed deaths from the day after a given country had cumulatively observed over 10 deaths (*d*_10_), given that the early stages of the epidemic in a given country might be dominated by infections that are not local. An exception was made in the case of small regions or countries with a low number of deaths, such as Iceland and La Rioja, in which observed deaths were included from the day of the first death (*d*_1_). Similarly, according to the original procedure [4], the initial infections of the model were assumed to be 30 days before this *d*_10_ (or *d*_1_) day, starting with 6 consecutive days with the same number of infections, which was left as a parameter to be optimized in the fitting procedure. The initial infections for the first 6 days and the *R*_0_ and *R*_t_ values after each intervention were defined as parameters to optimize in the model. Fitting was done in the probabilistic programming language Stan, using an adaptive Hamiltonian Monte Carlo (HMC) sampler. Eight chains for 4000 iterations, with 2000 iterations of warmup and a thinning factor 4 were run. Running of 200 sampling iterations with 100 warmup iterations yielded very similar results in most of the cases, suggesting that convergence was achieved early in the fitting process. See more details in the original description of the model [4]. The original code is available at https://github.com/ImperialCollegeLondon/covid19model/releases/tag/v1.0.

In the original study, parameters were estimated simultaneously for 11 countries, but here the model was fitted to the data from each country/region independently, since the effect of each intervention on *R*_t_ is not necessarily the same in all areas. To simplify the model, here the number of interventions were reduced, and the possibility of having an end date for any intervention was added. The inclusion of only one intervention was found to be sufficient to explain the data, while the addition of further intervention steps did not significantly improve the fitting.

### 2.4. Predictive Model from a Given Set of Parameters

**Figure 2:**
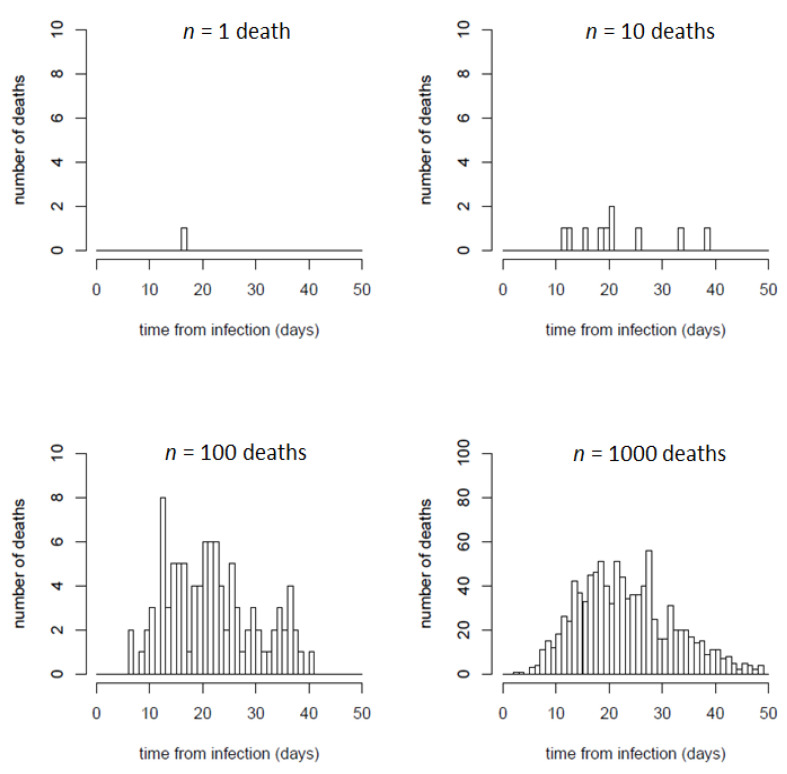
Examples of random discrete distributions of samples of *n* = 1, 10, 100, 1000 expected deaths, according to the infection-to-death (ITD) probability distribution used in this work. For samples of a small size, the distribution can be very different from the probability curve shown in Figure 1b, while for samples of a larger size (e.g., 100 or 1000 deaths), the discrete distributions get closer to the probability distribution curve.

The use of continuous serial interval and infection-to-death probability distributions to estimate the number of deaths from the new infections is needed for the efficiency of the fitting procedure, but in reality, the deaths derived from the newly infected people on a given day will be distributed in specific days in a discrete manner. The overall discrete distributions will expectedly follow the serial interval and infection-to-death probability distributions, but the number of deaths for each set of infections will happen in a single discrete distribution, which will be different each time, especially if the number of infections is small. In Figure 2 we can compare some random discrete distributions for expected deaths over time for samples of different sizes (*n* = 1, 10, 100, 1000) according to the continuous infection-to-death probability distributions used here vs. several discrete samples derived from the same infection-to-death distribution. As the sample size (*n*) increases, the discrete and continuous distributions tend to converge, but for small size samples, assuming continuous distribution might not represent a given instance.

Therefore, for a better visual comparison between the predictive results of our model and the set of reported deaths over time (discrete distribution), it is possible to generate instances of discrete distributions over time of the expected number of new infections and deaths computed with the parameters obtained during the fitting procedure (usually, from a set of parameters randomly selected among the ones obtained). These instances can be generated by randomly assigning every case (from the set of new infections or deaths to be distributed) to a specific day according to the serial interval and infection-to-death probability distributions.

### 2.5. Estimating Active Cases from Model Predictions

Basically, the expected number of recovered cases *RECOV_i_* in a given day *i* is a function of the number of the estimated new infections *I_j_* occurring in the previous *j =* 1…*i−*1 days, according to a previously calculated infection-to-recovery (*ITR*) probability distribution, after discounting the percentage of new infected cases with outcome of death from infection fatality ratio (IFR) (Equation (3)).

(3) $$RECOV_{i}=\overset{i-1}{\underset{j=1}{\sum }}I_{j}\cdot \left(1-IFR\right)\cdot ITR_{i-j}$$

The infection-to-recovery (*ITR*) probability distribution was modelled by adding up two independent distributions: (i) the infection-to-onset (incubation period) distribution, estimated as a Gamma probability density function with mean 5.1 days and a coefficient of variation 0.86; and (ii) the onset-to-recovery (time between onset and recovery) distribution, estimated as a Gamma probability density function with a mean of 24.7 days and a coefficient of variation 0.35 [7]. The resulting *ITR* probability distribution is shown in Figure 1c. The estimated active cases for a given day is calculated by subtracting the estimated cumulative number of recovered cases from the estimated cumulative number of cases for that day.

## 3. Results

### 3.1. Model Suggests a Significant Impact of Intervention Measures on Disease Transmission

The model was fitted (see Methods) to data from the countries with the highest number of reported cases in the EU/EEA and the UK as of 5 May 2020 (Spain, Italy, United Kingdom, Germany and France) (Table 1). They are actually the countries with the largest population. For comparison, the data for small countries and regions are also added, such as Iceland, and the Spanish region La Rioja, which have a comparable population (over 300,000 inhabitants) and show a high incidence of cases per 100,000 people. Actually, La Rioja is the region with highest number of detected cases relative to its population in Spain and probably in Europe. For comparison, the region in the United States with the highest level of incidence is New York City, in boroughs like The Bronx with 2667 detected cases per 100,000 as of 5 May 2020 [10].

**Figure 3:**
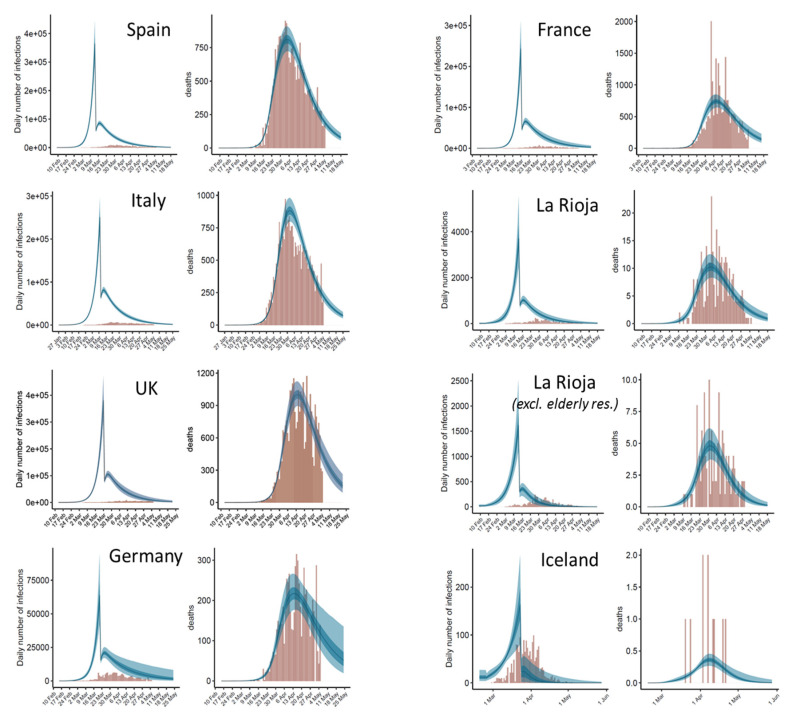
Estimates of infections and deaths over time after independent application of the model to each country and region. For each country, the left plot shows the predicted daily infections as compared with the reported ones, and the right plot shows the expected daily deaths as compared with the observed ones. Expected values are shown as blue bands: light blue (95% CI), dark blue (50% CI) and line (median). Observed values are shown as brown bars. The estimates of *R*_t_ before and after intervention resulting from the model are the ones in Table 3. In all cases, after intervention, *R*_t_ is significantly reduced to a value well below 1 and the number of new infections decreases. In the La Rioja second panel, daily observed deaths do not include those from elderly retirement homes, but daily reported cases are the total ones, since the daily reported cases outside elderly residences was not available for this work.

The study here focuses on the effect of the single most relevant intervention measure implemented by authorities (defined as “lockdown ordered” in a previous study [4]). For La Rioja, the same lockdown date was used as for Spain (14 March). For Iceland, the date of 24 March has been considered, when a nation-wide ban was enforced on public assemblies over 20, as well as the closure of bars and most public businesses. The dates of these interventions are shown in Table 2. After fitting the model for each country/region independently, the estimated number of daily infections and deaths derived from the model are shown in Figure 3, in comparison with the reported ones. The resulting reproduction number values derived from the model provide an estimation of the impact of the intervention measures on the transmission dynamics of the disease in each country (Table 3).

We can observe that the final *R*_t_ after major intervention is similar in all analyzed countries (except in Iceland and in La Rioja when elderly residences with reported cases were excluded), with mean values ranging from 0.57 (La Rioja region) to 0.71 (Germany), and an averaged value of 0.625. Interestingly, a recent calculation on the reproduction number (*R*) in Germany, estimated from a nowcasting approach on reported COVID-19 cases with illness onset up to three days before data closure, provides a current estimate of *R* = 0.71 (95% prediction interval: 0.59–0.82) [11], which is virtually the same as the one calculated here with the disease transmission model based on the reported deaths. Regarding the relative values of *R_t_* after intervention (in percentage relative to *R*_0_ before intervention), they ranged from 12.0% (Spain) to 20.7% (Italy). These values seem to depend not only on the effectiveness of the intervention measures, but also on the evolution of the disease prior to intervention, described by *R*_0_, which seems to be different in each country (Table 3). As a warning note, this effect in relative terms was the one assumed to be constant for the same type of intervention in the different countries in the original application of the model [4], an assumption that does not seem to be valid.

The model has also been applied by including additional interventions, such as the work lockdown period ordered in Spain and La Rioja region between 1 and 6 April 2020 (see Supplementary Materials Table S1), or the contention period around 7 March 2020 in La Rioja after a very localized outbreak in the city of Haro (data not shown), but no significant improvement of the model was found (Supplementary Materials Figure S1). The effect of an additional indirect intervention in Iceland was also modelled (on 3 April, a recently released mobile app to trace infections had almost 75,000 downloads, which might have had an impact on the reproduction number), with no significant changes (Supplementary Materials Table S1, Supplementary Materials Figure S1). In general, one would expect that a series of stepwise interventions will have a smoother effect on the reproduction number (and therefore on the number of new infections) than just a single strict intervention. One could also speculate whether other early measures taken in some countries have contributed to a decrease in their initial *R*_0_ values or to a delay in the initial number of infections at the early stage of the epidemic outbreak, but this is beyond this study.

It is interesting to analyze the results after applying the model to La Rioja data after excluding data in elderly retirement homes with reported cases (Figure 3, Table 3). As expected, the propagation of the disease in the elderly retirement homes and in the general population is very different. Outside these elderly retirement homes, the propagation rates decrease before and after intervention. It would be interesting to know whether the same effect can be seen in other countries.

### 3.2. Estimated Number of Total Infections and Active Cases

**Figure 4:**
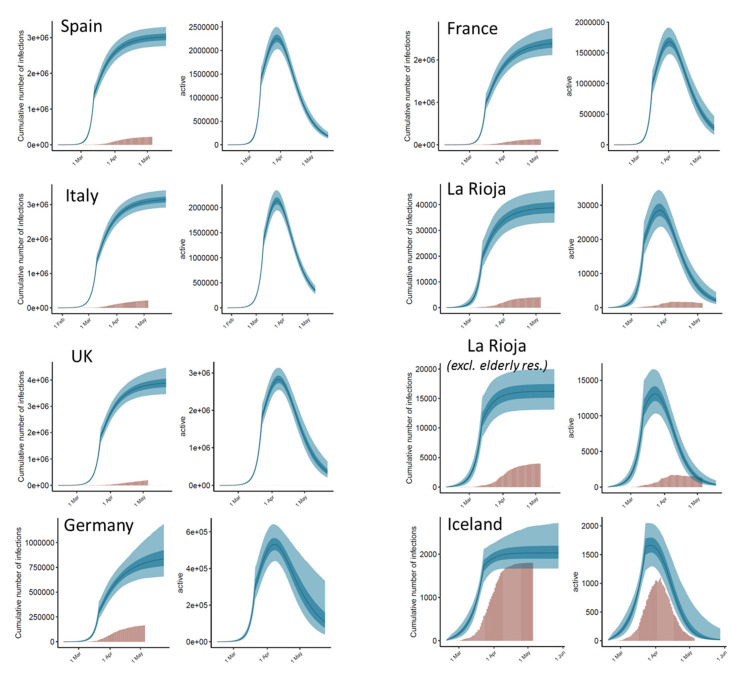
Estimated cumulative infections and active cases from an independent application of the model to each country and region. For each country, the left plot shows the estimated cumulative infections for each day as compared with the reported ones, and the right plot shows the expected active cases for each day (in some countries, they are compared with the reported ones). Expected values are shown as blue bands: light blue (95% CI), dark blue (50% CI) and line (median). Observed values are shown as brown bars. The estimates of *R*_t_ before and after intervention resulting from the model are the ones in Table 3. In all cases, after intervention, *R*_t_ is significantly reduced to a value well below 1 and the number of new infections decreases. In the La Rioja second panel, estimates are derived from observed deaths after excluding those from elderly retirement homes, but accumulated infections and active cases over time are the total ones, since the daily cases excluding elderly retirement homes was not available for this work.

From the values obtained by the model, we can estimate the daily cumulative number of infections. The evolution of the predicted cumulative number of infections in comparison with the detected cases over time can be seen in Figure 4, with the detailed data per country as of 5 May 2020 in Table 4. The highest detection rates (that is, total detected cases with respect to the total predicted ones) are found in Germany (21%) and Iceland (54%). These are actually countries with a high number of tests performed relative to their population. In Germany, there were a reported 30.4 tests per thousand people as of 22 April 2020 [12], one of the highest rates in Europe. In Iceland there were a reported 151.2 tests per thousand people as of 5 May 2020, the highest rate in Europe [13]. In other territories, the total number of predicted cases is much higher than the reported ones, with detection rates that range from 5% (UK) to 10% (La Rioja). Interestingly, the detection rate in La Rioja is better than in Spain (7%). Moreover, when data from elderly retirement homes with reported cases are excluded, the detection rate in the rest of the population in La Rioja is much larger (18%). This is consistent with the fact that La Rioja was the Spanish region with the highest number of PCR tests per thousand people (56.9, as of 27 April 2020; as compared to 22.0 in Spain) [14].

From the model results, we can also estimate the number of daily active cases per country (Figure 4). For comparison, the reported number of active cases in La Rioja [15] and in Iceland [13] are shown. In La Rioja, there were a reported 1257 active cases as of 5 May 2020. As indicated in Table 4, the model estimated a median value of 4746 for the same date (95% CI: 2821–8088), which indicates a detection rate of 27%. When data from elderly retirement homes were excluded, the model estimated a median value of 945 active cases (95% CI: 464–2059). Considering that the reported 1257 active case as of 5 May include cases from elderly residences (e.g., 112 out of the 741 active cases as of 15 May were from elderly retirement homes [15]), this indicates a detection rate of active cases close to 100%. In Iceland, there were a reported 32 active cases as of 5 May 2020, while our model predicted a median value of 141 active cases for the same date (95% CI: 78–605) (Table 4), indicating a detection rate of 23% (which is lower than the general detection rate for the total cumulative cases).

### 3.3. Predicting Discrete Distributions of Infections and Deaths

**Figure 5:**
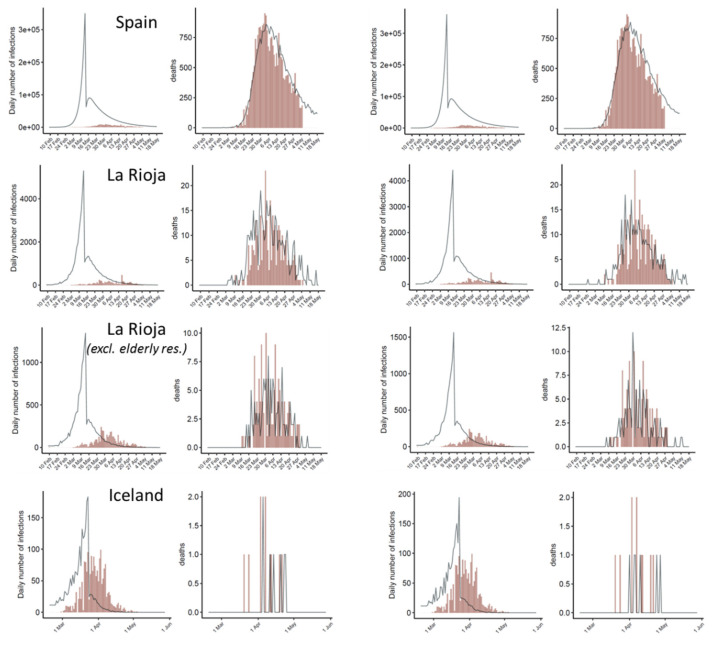
Instances of discrete distributions of the estimated number of daily infections and deaths randomly sampled according to the serial interval and infection-to-death probability distributions, based on randomly selected sets of reproduction numbers and initial infections among the ones obtained by the fitted model (black lines), as compared to the reported data (brown bars). Each row represents a country, with two instances of discrete distributions. When the number of daily infections and deaths is large (e.g., Spain), the predicted discrete distributions are closer to the continuous ones (Figure 3), but when these numbers are smaller (e.g., La Rioja and Iceland), the discrete distributions resemble better the rough distribution of reported data over time. In the La Rioja second panel, observed deaths do not include those from elderly retirement homes, but daily reported cases are the total ones, since daily cases excluding elderly retirement homes was not available for this work.

A continuous distribution of the expected new infected cases and deaths provides a smooth curve that is suitable for parameter optimization. However, in reality, the infections and deaths are obviously distributed in a discrete manner. For better visualization of the predicted data, random discrete distributions of the expected infections and deaths have been generated, according to the continuous probability distributions based on randomly selected parameters among the ones resulting from the model (see Section 2.4). Figure 5 shows some random discrete samples of the predicted infections and deaths, which resemble better the noisy distribution of the reported data, especially in small countries and regions.

### 3.4. The Reliability of the Predictions Depends on the Stage of the Epidemic Outbreak

**Figure 6:**
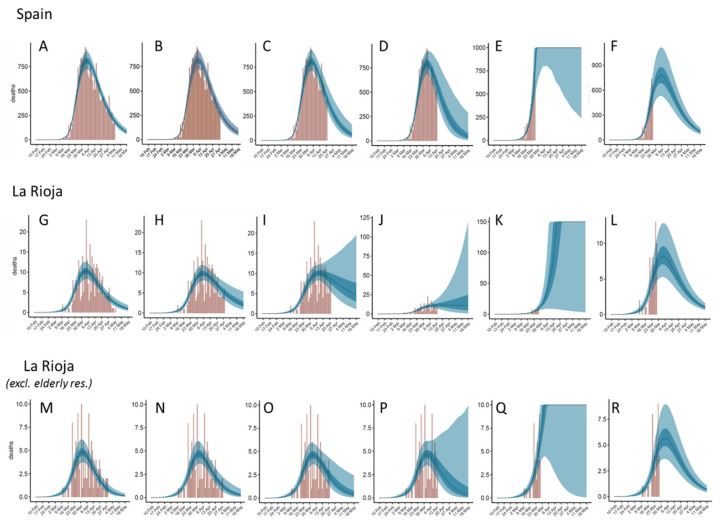
Estimated deaths for Spain (upper row) and La Rioja (total data: middle row; excluding elderly residences: bottom row) derived from the model (**A**,**G**,**M**) in comparison to the predictions obtained after removing the last week (**B**,**H**,**N**), last 2 weeks (**C**,**I**,**O**), last 3 weeks of data (**D**,**J**,**P**) or keeping data just up to 1 week to the peak (**E**,**K**,**Q**), the latter also after keeping constant *R_t_* = 0.625 (**F**,**L**,**R**).

The credible intervals of the parameters and estimated data provided by the model help to assess the robustness of the predictions. In the graphical plots of estimated daily cases and deaths (Figure 3), we can visualize the predictions for a few days beyond the last day of data (5 May 2020). In general, in countries where more time has passed since the peak of the outbreak (e.g., Spain, Italy), the variability in the estimated data beyond the last day of data is smaller than in countries in which fewer days have passed from the time of the peak (e.g., Germany, UK). As a validation test, in order to evaluate the capabilities of the model to predict the evolution of the disease beyond current date, it has been fitted again to Spain and La Rioja data (with and without elderly residences cases), after removing the reported deaths corresponding to the last week, the last two weeks or the last three weeks of data in Spain and La Rioja (Figure 6B–D,H–J,N–P).

Table 5 shows the reproduction numbers, and the estimated number of deaths for the last week of data (29 April–5 May) obtained by the model when different sets of dates are removed. In general, we can observe that the more dates that are removed and the fewer days left after the peak of the outbreak, the more uncertain the predictions get. Indeed, the predicted *R_t_* values obtained after removing larger sets of dates increasingly deviate from the ones obtained with the entire set of dates. Similarly, the number of deaths predicted for the last week of data increasingly deviate from the reported ones when larger sets of dates are removed. However, the response of the model to the removal of data is different in the two cases analyzed here. In the case of Spain, the mean value for the predicted deaths in the last week does not dramatically deviate from the real one even when three weeks of data are removed, although the uncertainty increases (the 95% CI range increases with respect to the ones obtained with the entire set of dates). We can safely say that three weeks ago the model would have been able to reasonably predict today’s situation in Spain (as of 5 May 2020). In the case of La Rioja, the predictions get worse much faster upon removal of data, with larger deviations of *R_t_* and a worse prediction of deaths for the last week of reported data. Perhaps this different behavior of the model with shorter data is related to specific features of the disease evolution in each territory, or maybe the reasons can be found in the differences in sample size between Spain and La Rioja (i.e., removing dates from regions with an already small number of reported cases can introduce a larger uncertainty).

It is interesting to evaluate the predictive capabilities of the model when using data just up to one week before the peak of the outbreak. In this case (Figure 6E,K,Q), predictions are much more uncertain and completely wrong. As we can see in Table 5, while the *R*_0_ values are not dramatically different, the resulting *R_t_* values are completely wrong, which indicates that the model cannot estimate the impact of the intervention on the reproduction number before achieving the peak of the outbreak. This was actually the situation of the original study on 11 countries on 28 March [4], when the peak of the outbreak was still >1 week away in the majority of analyzed countries. Consistent with the validation test here, in those conditions (>1 week before the peak) the model underestimated the impact of the different intervention measures and predicted a much higher number of infections and death than the reported ones in the following days.

Fortunately, as mentioned above (see Table 3), the values obtained for *R_t_* (after a major intervention) seem to be quite consistent across all countries (except Iceland), with mean values between 0.57 and 0.71, and an average value of 0.625. Considering this, the model was fitted again to the reduced set of data (up to one week before the peak), but this time assuming a locked value of *R_t_* = 0.625. Remarkably, in these conditions, the predictions are actually quite good (Figure 6F,L,R) and are indeed comparable to those obtained with the entire set of data. This validation test suggests that in cases in which the epidemic outbreak has not yet clearly passed its peak, especially when the available data is noisy (e.g., small sample size), it could be a better option to apply the model using a guess value of *R_t_* = 0.625 rather than trying to predict such an *R_t_* value by fitting.

### 3.5. Modelling Long-Term Disease Progression in Different Scenarios

**Figure 7:**
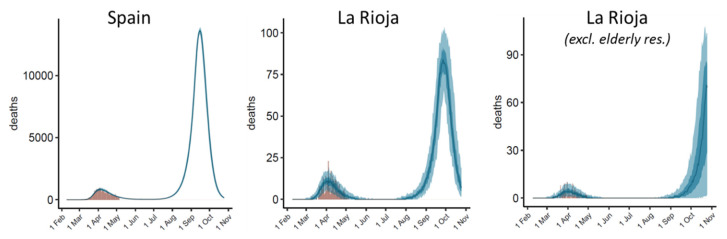
Forecast for daily deaths in Spain and La Rioja over the upcoming months (total data, after excluding data from elderly residences) assuming *R*_t_ = 1.0 between 11 May and 22 June 2020 and *R*_t_ = 1.8 afterwards.

The above described model is a useful tool to predict the evolution of the disease in each country or community. In these moments, it is urgent to evaluate the possible outcome of the planned changes in current transmission control measures. For instance, in Spain (including La Rioja), a gradual return to the situation prior to intervention is proposed starting on 11 May 2020 in most regions, in which meetings of up to 10 people will be allowed and bar terraces will be opened (with some limitations), and ending in 22 June 2020, when some regions will be able to return to close to pre-pandemic conditions, allowing sports and cultural shows (with some conditions), as well as national travel. We can devise different scenarios regarding the potential effect of this on the reproduction number due to the gradual removal of lockdown measures on these key dates. Some options are: (i) no changes in *R*_t_ (very unlikely); (ii) slight increment to *R*_t_ = 0.71 similar to the current situation in Germany (also quite unlikely given the activities that are planned to be allowed); (iii) further increment to *R*_t_ = 1.0, which implies a doubling of the number of patients actively transmitting the disease (this could be a likely scenario for the period between 11 May and 22 June); or (iv) a much larger increment to *R*_t_ = 1.8, similar to the situation in Iceland at the beginning of the epidemic, e.g., normal activities allowed but with extensive testing and isolation of detected infections (this is a likely scenario after 22 June). All these options have been considered for Spain and La Rioja, and the results are shown in Supplementary Materials Figures S2 (Spain), S3 (La Rioja) and S4 (La Rioja, excluding data from elderly residences). There is a further possible scenario that we can evaluate, which is a possible full return to the pre-pandemic period in 1 September 2020, with open schools, usual sports and cultural shows, etc., so this variable has also been considered in Supplementary Materials Figures S5 (Spain), S6 (La Rioja) and S7 (La Rioja, excluding data from elderly residences). We can see that in some of these scenarios, the possibility of a new epidemic outbreak is very clear. Figure 7 shows in more detail the predicted evolution for Spain in La Rioja (with and without elderly retirement home data) in a likely scenario, with *R*_t_ = 1.0 between 11 May and 22 June and *R*_t_ = 1.8 for the period afterwards. Assuming these reproduction numbers, we can foresee an outbreak in September/October. Obviously, the model does not consider possible intervention measures to be taken to limit the impact of this hypothetical outbreak, which might depend on how early the potential new infections could be detected.

The model considers that all new infected cases have the same probability of infecting new people (which is given by the *SI* distribution and the estimated *R*_t_ in Equation (1)). However, in a situation such as the one in La Rioja where the cases inside the elderly retirement homes are reasonably isolated from the rest of the population and outside elderly residences the virtual totality of active cases seem to be detected and is thus unlikely to induce new infections, the effective *R*_t_ values on the analyzed dates could be much lower than those in the hypothetical scenarios discussed here, which would suggest a more optimistic situation in the upcoming months. In any case, after 22 June, with travels between provinces in Spain allowed, it will be essential to be able to detect any new focus of infection that might induce a sudden outbreak. For comparison, assuming the same scenario in Iceland (*R*_t_ = 1.0 between 11 May and 22 June 2020 and *R*_t_ = 1.8 afterwards), no outbreak is predicted in the studied period, even when assuming a full return to pre-pandemic conditions on 1 September 2020 (data not shown). The model can thus be useful for evaluating the long-term impact of the implementation and/or removal of intervention measures on the disease evolution in a given country or region.

## 4. Discussion

The model used here depends strongly on the serial interval and infection-to-death probability distributions, which were based on data from the early epidemic outbreak in China. Future updates of the model could benefit from the use of other available probability distributions based on data from patients from different regions [16]. In some reported models, the incubation period averages around 5 days (CI 95% 2–14), with a median time delay of 13–17 days from illness onset to death, depending on the type of truncation [17]. In another study, using the most reliable data among their sets and including patients from Germany and South Korea, the median serial interval was estimated at 4.6 days (95% CI: 3.5–5.9) [18]. A recently proposed model of SARS-CoV-2 transmission assumed a latent period of 4.6 days and an infectious period of 5 days, informed by the best-fit values for other betacoronaviruses [2]. Finally, a recent study based on patients from countries and regions outside of Hubei province, China, estimated the median incubation period to be 5.1 days (95% CI 4.5–5.8) [19], similar to the value used here. In any case, serial interval and infection-to-death probability distributions will need to be updated by using epidemiological data from new countries and regions where the virus is expanding. Another parameter that can strongly affect the estimated number of infections is the IFR value. As mentioned above, there are several estimates for IFR based on available data [6,7,8] and on mathematical models [20].

A major limitation of the current model is related to the fact that *R* is actually a dynamic parameter that may change over time and take different values in each of the communities forming a country. However, here *R* is assumed to be constant during long periods of time, changing only upon a specific intervention. While this assumption seems to be adequate to estimate abrupt changes in *R* after major interventions, in many situations *R* can change over time in a more subtle way, for instance, it could depend on local actions such as disease transmission control in specific communities (e.g., elderly residences, hospitals) or on behaviors changing over time, like the self-awareness of the population. This seems to be the case in Iceland, in which *R*_0_ is smaller than in other countries, probably because of a better control of the first detected cases thanks to a higher detection rate. Inclusion of a more dynamic *R_t_* may lead to significant improvements of the model, but also to increased noise in the fitting process unless more data can be considered.

As mentioned in Section 2.2, the entire population was assumed to be susceptible to infection during model fitting, a reasonable approximation in the current study given that only a small proportion of the population in the analyzed countries/regions was infected during this first outbreak. However, when modelling future possible outbreaks, we should take into consideration that part of the population might have been immunized after first infection. Indeed, infected cases were already assumed to be protected from further infection in the long-term predictions shown here (Section 3.5). Now, for updated versions of the model, it would be important to include this possibility during model fitting as well. In addition, future updates could include further restraints on the fraction of the population susceptible to infection, for instance, by assuming that detected cases are not likely to be infective since they should be isolated in quarantine, or by separately considering isolated communities, such as elderly retirement homes, as has already been shown here.

Regarding the size of the data sample, while larger sets of data in entire countries show softer distribution curves of reported infections and deaths, there is also an implicit difficulty in describing the disease evolution with a single model, because the transmission dynamics in a country are usually formed by the disease evolution in different communities. This seems to be the case in Italy, in which the fitted model expects a single peak after a major intervention, while the distribution of reported deaths over time seem to have a shoulder after the main peak (Figure 3). This might indicate spreading from the initial focus to other regions, which can largely affect the transmission dynamics in Italy [21]. While this geographical transmission is not explicitly considered in the model, its application can be a complement to other studies aiming to understand the evolution of the disease in time and space. For instance, our model estimates a total of 396 infections (95% CI: 153–819) in Spain between 9 and 14 February 2020. The model cannot distinguish whether these were local infections or whether they were externally acquired, but the number is consistent with recent studies on the spread of disease in Spain in mid-February, based on phylogenetic studies using SARS-Cov-2 whole-genome sequencing data, which estimated the origin of two SARS-Cov-2 clusters in Spain around 14 and 18 February, 2020 [22].

## 5. Conclusions

A Bayesian model of disease transmission inferred from reported deaths, previously developed by researchers at Imperial College London, has been updated and independently fitted to European countries and regions with a high number of reported cases, or a high incidence related to their population. The model indicates that major intervention measures have a similar impact on the disease transmission in most of these countries and provides realistic estimates of the total number of infections, active cases and future outcomes. The model can also be useful to help plan changes in the implementation of control measures, but the reliability of the long-term predictions may depend on the moment of the epidemic outbreak.

## Figures and Tables

**Table 1:** Reported COVID-19 cases per country/region (updated 5 May 2020).

Country/Region	Total Detected Cases	Cases per 100 k People	Total Reported Deaths
Spain	219,329	469	25,613
Italy	211,938	351	29,079
UK	190,584	287	28,734
Germany	163,860	198	6831
France	131,863	197	25,201
La Rioja (Spain)	3969	1256	336
La Rioja (Spain) ^1^	2988	952	143
Iceland	1799	509	10

^1^ Excluding data from elderly retirement homes with reported cases. Reported deaths (excluding those in elderly retirement homes) correspond to 5 May 2020, but detected cases outside elderly retirement homes have been extrapolated from data on 15 May 2020.

**Table 2:** Model parameters (intervention dates and IFR) used in this work.

Country/Region	Time of Intervention	Days from First 100 Cases to Intervention Time	IFR
Spain	14 March	11	0.926%
Italy	11 March	16	1.090%
UK	24 March	18	0.919%
Germany	22 March	20	1.093%
France	17 March	15	1.153%
La Rioja	14 March	5	0.926%
Iceland	24 March	8	0.556%

**Table 3:** Estimated reproduction numbers (before and after intervention) and initial number of infections resulting from the model (shown mean values, with 95% credible interval).

Country/Region	*R*_0_	*R*_t_ after Intervention	Estimated Infections in First 6 Days
Spain	4.82 (4.18–5.51)	0.58 (0.52–0.65)	396 (153–819)
Italy	3.14 (2.93–3.38)	0.65 (0.60–0.70)	623 (370–964)
UK	3.60 (3.26–3.95)	0.60 (0.50–0.70)	749 (396–1276)
Germany	3.68 (2.91–4.57)	0.71 (0.54–0.89)	314 (70–876)
France	4.47 (3.93–5.06)	0.64 (0.55–0.74)	113 (43–242)
La Rioja	3.29 (2.41–4.48)	0.57 (0.45–0.70)	72 (8–239)
La Rioja ^1^	2.55 (2.06–3.34)	0.41 (0.21–0.59)	123 (27–279)
Iceland	1.84 (1.37–2.38)	0.26 (0.01–0.69)	75 (24–162)

^1^ Excluding data from elderly retirement homes with reported cases.

**Table 4:** Estimated cumulative infections as of 5 May 2020 (shown median values with 95% credible interval).

Country/Region	Estimated Total Infections	Detection Rate	% Population Infected	Estimated Active Cases
Spain	2990K (2742K–3269K)	7.3% (6.7–8.0%)	6.4% (5.9–7.0%)	415K (329K–538K)
Italy	3094K (2868K–3358K)	6.9% (6.3–7.4%)	5.1% (4.7–5.6%)	445K (365K–551K)
UK	3800K (3406K–4292K)	5.0% (4.4–5.6%)	5.6% (5.0–6.3%)	998K (739K–1369K)
Germany	793K (641K–1024K)	20.6% (16.0–25.6%)	0.9% (0.8–1.2%)	245K (142K–451K)
France	2351K (2089K–2693K)	5.6% (4.9–6.3%)	3.6% (3.2–4.1%)	482K (341K–715K)
La Rioja	38,505 (32,850–45,155)	10.3% (8.8–12.1%)	12.2% (10.4–14.3%)	4746 (2821–8088)
La Rioja ^1^	16,205 (13,095–19,972)	18.4% (15.0–22.8%)	5.2% (4.2–6.4%)	945 (464–2059)
Iceland	2029 (1669–2647)	88.7% (68.0–100%)	0.6% (0.5–0.7%)	141 (78–605)

^1^ Excluding data from elderly retirement homes with reported cases.

**Table 5:** Reproduction number and expected deaths during the last week of reported data (29 April–5 May) after removing different sets of dates from the model.

Country/Region	Forecast Start Time	*R*_0_	*R*_t_	Deaths 29 April–5 May
Spain				1791 (real)
	5 May (last day, original model)	4.82 (4.18–5.51)	0.58 (0.52–0.65)	1653 (1386–1980)
	28 April (1 week to last)	4.87 (4.20–5.63)	0.57 (0.48–0.65)	1562 (1178–2048)
	21 April (2 weeks to last)	4.88 (4.13–5.69)	0.54 (0.40–0.70)	1470 (853–2466)
	14 April (3 weeks to last)	4.90 (4.03–5.84)	0.50 (0.23–0.78)	1344 (430–3435)
	27 March (1 week to peak)	4.08 (3.28–5.06)	2.99 (0.78–4.37)	off-limits (2972–off)
	27 March (1 week to peak) locked *R*_t_	4.56 (3.57–5.66)	*0.625*	1864 (1223–2666)
La Rioja				10 (real)
	5 May (last day, original model)	3.29 (2.41–4.48)	0.57 (0.45–0.70)	19 (13–28])
	28 April (1 week to last)	3.00 (2.33–4.17)	0.71 (0.55-0.88)	31 (18–50)
	21 April (2 weeks to last)	2.81 (2.24–3.83)	0.84 (0.61–1.10)	52 (22–111)
	14 April (3 weeks to last)	2.72 (2.17–3.74)	0.98 (0.54–1.44)	108 (16–358)
	28 March (1 week to peak)	2.76 (2.22–3.69)	2.29 (0.80–3.35)	off-limits (37-off)
	28 March (1 week to peak) locked *R*_t_	2.86 (2.22–4.00)	*0.625*	19 (11–29)
La Rioja (excluding elderly residences)				2 (real)
	5 May (last day, original model)	2.55 (2.06–3.34)	0.41 (0.21–0.59)	5 (2–8)
	28 April (1 week to last)	2.46 (1.97–3.15)	0.51 (0.29–0.72)	7 (3–13)
	21 April (2 weeks to last)	2.45 (1.93–3.14)	0.58 (0.28–0.87)	10 (3–24)
	14 April (3 weeks to last)	2.44 (1.87–3.12)	0.58 (0.14–1.10)	13 (2–57)
	28 March (1 week to peak)	2.53 (2.04–3.34)	1.88 (0.32–2.88)	off-limits (3–off)
	28 March (1 week to peak) locked *R*_t_	2.61 (2.06–3.59)	*0.625*	13 (8–20)

## Supplementary Materials

The following are available online at https://www.mdpi.com/2077-0383/9/6/1825/s1, Table S1: Parameters (reproduction number and initial infections) obtained by using additional interventions in the modeling, Figure S1: Estimates of infections and deaths obtained by including additional interventions dates in the model, Figure S2: Forecast for daily deaths in Spain over the upcoming months in different scenarios, Figure S3: Forecast for daily deaths in La Rioja over the upcoming months in different scenarios, Figure S4: Forecast for daily deaths in La Rioja (excluding data from elderly retirement homes with reported cases) over the upcoming months in different scenarios, Figure S5: Forecast for daily deaths in Spain over the upcoming months in different scenarios, assuming full return to pre-pandemic period on 1 September 2020, Figure S6: Forecast for daily deaths in La Rioja over the upcoming months in different scenarios, assuming full return to pre-pandemic period on 1 September 2020, Figure S7: Forecast for daily deaths in La Rioja (after excluding data from elderly retirement homes with reported cases) over the upcoming months in different scenarios, assuming full return to pre-pandemic period on 1 September 2020.

## References

[1] World Health Organization Coronavirus Disease 2019 (COVID-19) Situation Report—106 (WHO, 2020). https://www.who.int/docs/default-source/coronaviruse/situation-reports/20200505covid-19-sitrep-106.pdf?sfvrsn=47090f63_2.

[2] Kissler S.M., Tedijanto C., Goldstein E., Grad Y.H., Lipsitch M. Projecting the transmission dynamics of SARS-CoV-2 through the postpandemic period. Science.

[3] Li R., Pei S., Chen B., Song Y., Zhang T., Yang W., Shaman J. Substantial undocumented infection facilitates the rapid dissemination of novel coronavirus (SARS-CoV2). Science.

[4] Flaxman S., Mishra S., Gandy A., Unwin H.J.T., Coupland H., Mellan T., Zhu H., Berah T., Eaton J., Perez Guzman P. (2020) Estimating the Number of Infections and the Impact of Non-Pharmaceutical Interventions on COVID-19 in 11 European Countries. https://www.imperial.ac.uk/mrc-global-infectious-disease-analysis/covid-19/report-13-europe-npi-impact/.

[5] Google COVID-19 Community Mobility Reports. https://www.google.com/covid19/mobility/.

[6] Ferguson N., Laydon D., Nedjati-Gilani G., Imai N., Ainslie K., Baguelin M., Bhatia S., Boonyasiri A., Cucunubá Z., Cuomo-Dannenburg G. (2020) Impact of Non-Pharmaceutical Interventions (NPIs) to Reduce COVID-19 Mortality and Healthcare Demand. https://www.imperial.ac.uk/mrc-global-infectious-disease-analysis/news--wuhan-coronavirus/report-9-impact-of-npis-on-covid-19/.

[7] Verity R., Okell L.C., Dorigatti I., Winskill P., Whittaker C., Imai N., Cuomo-Dannenburg G., Thompson H., Walker P.G., Fu H. Estimates of the severity of coronavirus disease 2019: A model-based analysis. Lancet Infect. Dis..

[8] Lourenço J., Paton R., Ghafari M., Kraemer M., Thompson C., Simmonds P., Klenerman P., Gupta S. (2020). Fundamental principles of epidemic spread highlight the immediate need for large-scale serological surveys to assess the stage of the SARS-CoV-2 epidemic. medRxiv.

[9] Oke J., Heneghan C. (2020). Global Covid-10 Case Fatality Rates. https://www.cebm.net/covid-19/global-covid-19-case-fatality-rates/.

[10] City of New York. COVID-19: Data. https://www1.nyc.gov/site/doh/covid/covid-19-data.page.

[11] Robert Koch Institute COVID-19 Daily Situation Report 5 May 2020. https://www.rki.de/DE/Content/InfAZ/N/Neuartiges_Coronavirus/Situationsberichte/2020-05-05-en.pdf?__blob=publicationFile.

[12] Robert Koch Institute COVID-19 Daily Situation Report 22 April 2020. https://www.rki.de/DE/Content/InfAZ/N/Neuartiges_Coronavirus/Situationsberichte/2020-04-22-en.pdf?__blob=publicationFile.

[13] Directorate of Health and the Department of Civil Protection and Emergency Management. https://www.covid.is/data.

[14] Spanish Health Ministry Press Release 27 April 2020. https://www.mscbs.gob.es/en/gabinete/notasPrensa.do?metodo=detalle&id=4883.

[15] Gobierno de La Rioja https://actualidad.larioja.org/coronavirus/datos.

[16] He X., Lau E.H.Y., Wu P., Deng X., Wang J., Hao X., Lau Y., Wong J.Y., Guan Y., Tan X. (2020). Temporal dynamics in viral shedding and transmissibility of COVID-19. medRxiv.

[17] Linton N.M., Kobayashi T., Yang Y., Hayashi K., Akhmetzhanov A.R., Jung S.M., Yuan B., Kinoshita R., Nishiura H. (2020). Incubation Period and Other Epidemiological Characteristics of 2019 Novel Coronavirus Infections with Right Truncation: A Statistical Analysis of Publicly Available Case Data. J. Clin. Med..

[18] Nishiura H., Lintona N.M., Akhmetzhanov A.R. (2020). Serial interval of novel coronavirus (COVID-19) infections. Int. J. Inf. Dis..

[19] Lauer S.A., Grantz K.H., Bi Q., Jones F.K., Zheng Q., Meredith H.R., Azman A.S., Reich N.G., Lessler J. The Incubation Period of Coronavirus Disease 2019 (COVID-19) From Publicly Reported Confirmed Cases: Estimation and Application. Ann. Int. Med..

[20] Bastolla U. (2020). How lethal is the novel coronavirus, and how many undetected cases there are? The importance of being tested. medRxiv.

[21] Gatto M., Bertuzzo E., Mari L., Miccoli S., Carraro L., Casagrandi R., Rinaldo A. (2020). Spread and dynamics of the COVID-19 epidemic in Italy: Effects of emergency containment measures. Proc. Natl. Acad. Sci. USA.

[22] Díez-Fuertes F., Caballero M.I., Monzón S., Jiménez P., Varona S., Cuesta I., Zaballos Á., Thomson M.M., Jiménez M., Pérez J.G. (2020). Phylodynamics of SARS-CoV-2 transmission in Spain. bioRxiv.

