# Supplementary material for: Modelling the Evolution of COVID-19 in High-Incidence European Countries and Regions: Estimated Number of Infections and Impact of Past and Future Intervention Measures
Source: J Clin Med. 2020 Jun 11;9(6):1825. doi: 10.3390/jcm9061825 (PMC7355559; doi:10.3390/jcm9061825)
Supplement: Supplementary file 1 [file jcm-09-01825-s001.pdf]

SUPPLEMENTARY DATA

Table S1. Parameters (reproduction number and initial infections) obtained by using additional interventions in the modeling

Country / region	Mean R_o (95% credible interval)	Interventions	Mean R_t after intervention (95% credible interval)	Estimated infections in first 6 days (95% credible interval)
Spain	4.80 (4.15-5.51)	14 Mar	0.61 (0.53-0.70)	401 (154-852)
		1-6 Apr	0.46 (0.10-0.62)	
La Rioja	3.22 (2.38-4.37)	14 Mar	0.63 (0.47-0.80)	78 (9-249)
		1-6 Apr	0.33 (0.02-0.62)	
Iceland	1.82 (1.31-2.38)	24 Mar	0.34 (0.02-1.05)	78 (25-171)
		3 Apr	0.20 (0.01-0.61)	

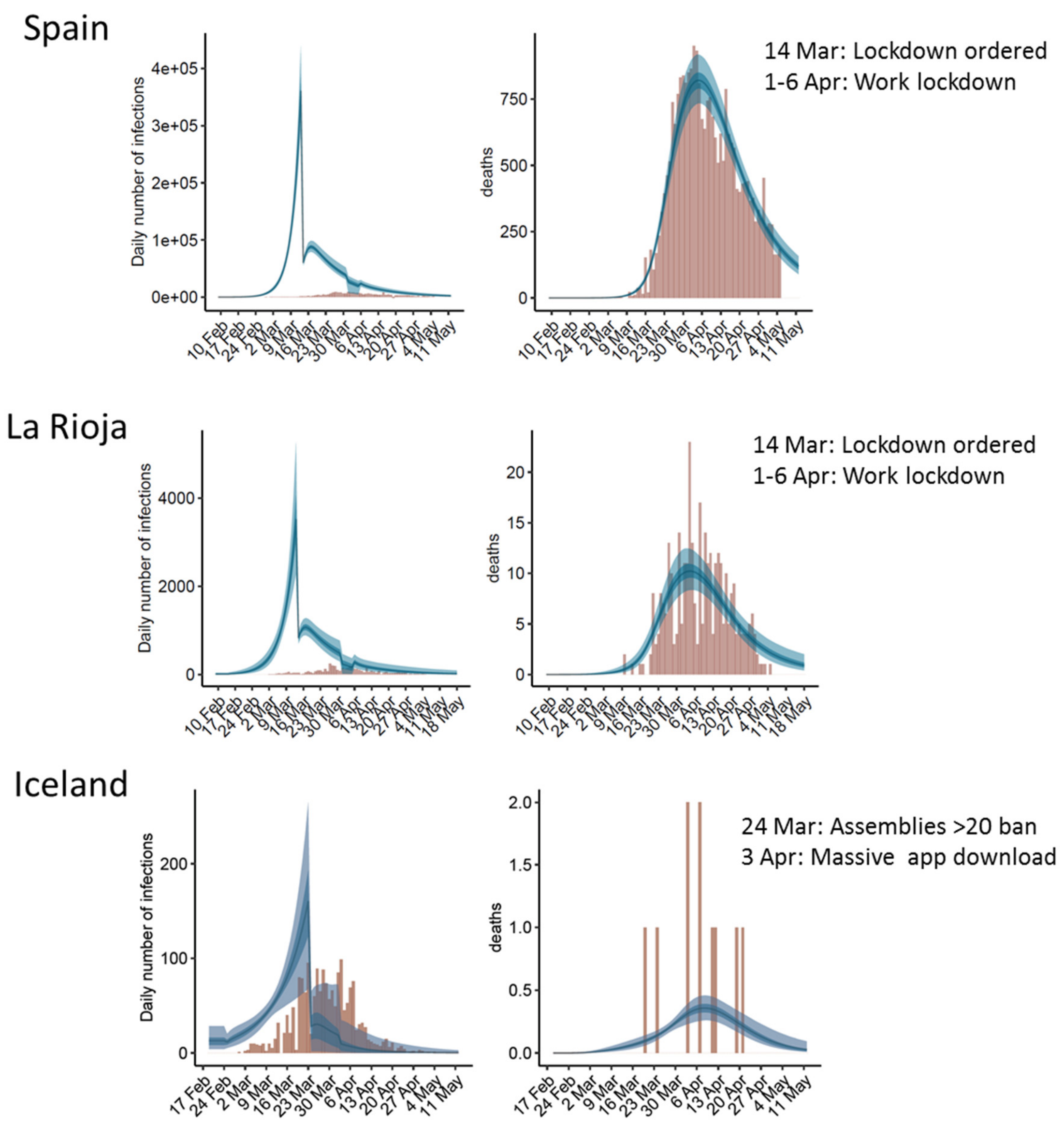

Figure S1. Estimates of infections and deaths obtained by including additional interventions dates in the model (Table S1).

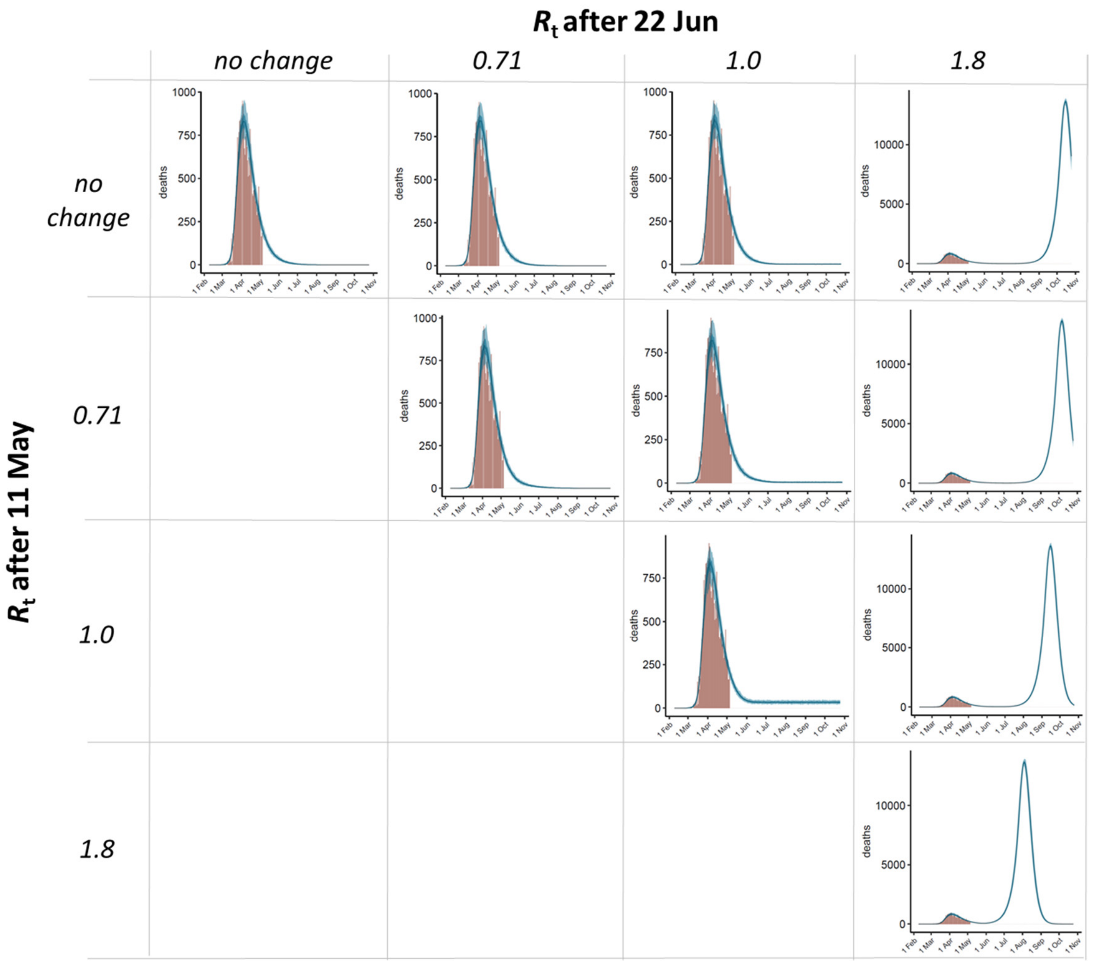

Figure S2. Forecast for daily deaths in Spain over the upcoming months in different scenarios. Blue bands (light blue 95% CI; dark blue 50% CI) represent the estimated daily deaths over time, computed as 100 discrete random distributions from sets of parameters obtained by the model (R_0, R_t after main intervention, and initial number of infections; Table 3), for different scenarios depending on possible changes in R_t after the proposed modifications in the intervention measures in two key dates (11 May and 22 June). Reported deaths to date are represented as brown bars.

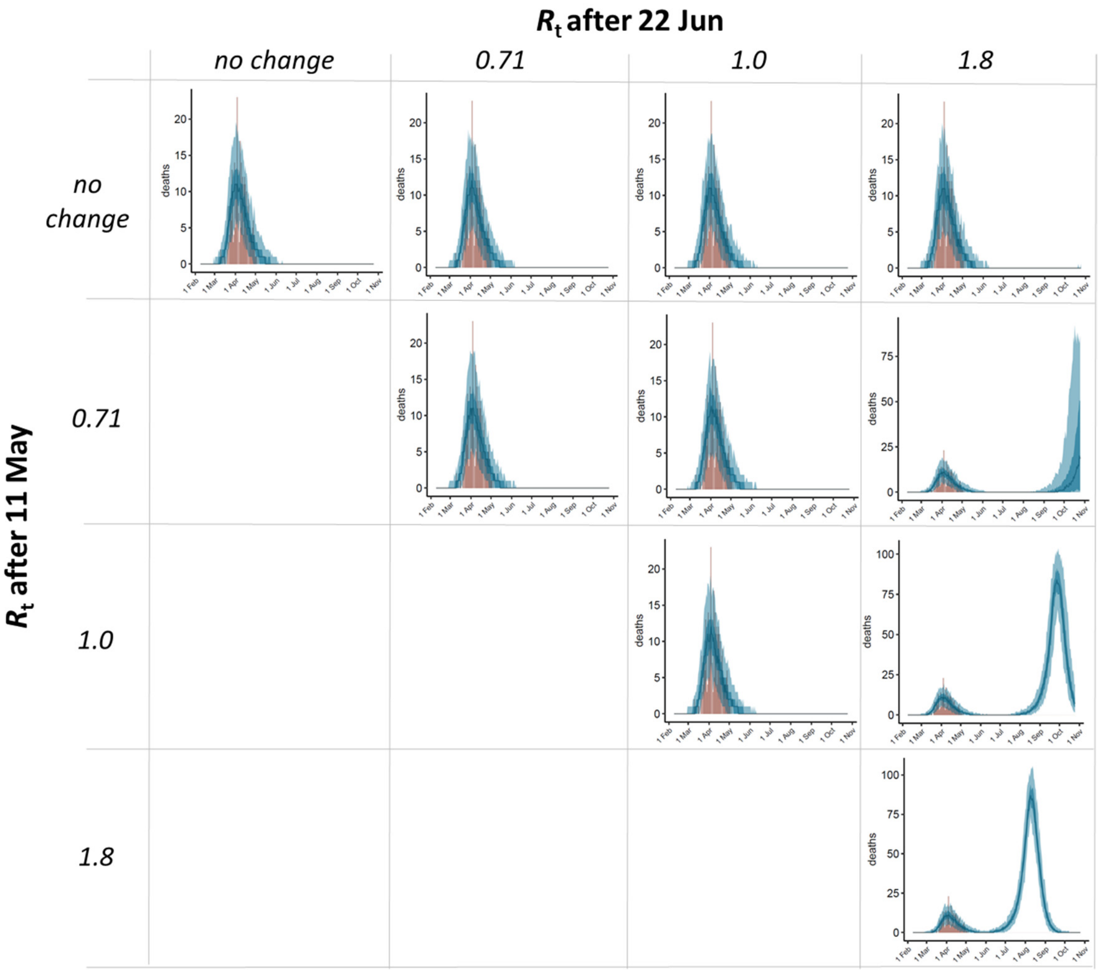

Figure S3. Forecast for daily deaths in La Rioja over the upcoming months in different scenarios. Blue bands (light blue 95% CI; dark blue 50% CI) represent the estimated daily deaths over time, computed as 100 discrete random distributions from sets of parameters obtained by the model (R_0, R_t after main intervention, and initial number of infections; Table 3), for different scenarios depending on possible changes in R_t after the proposed modifications in the intervention measures in two key dates (11 May and 22 June). Reported deaths to date are represented as brown bars.

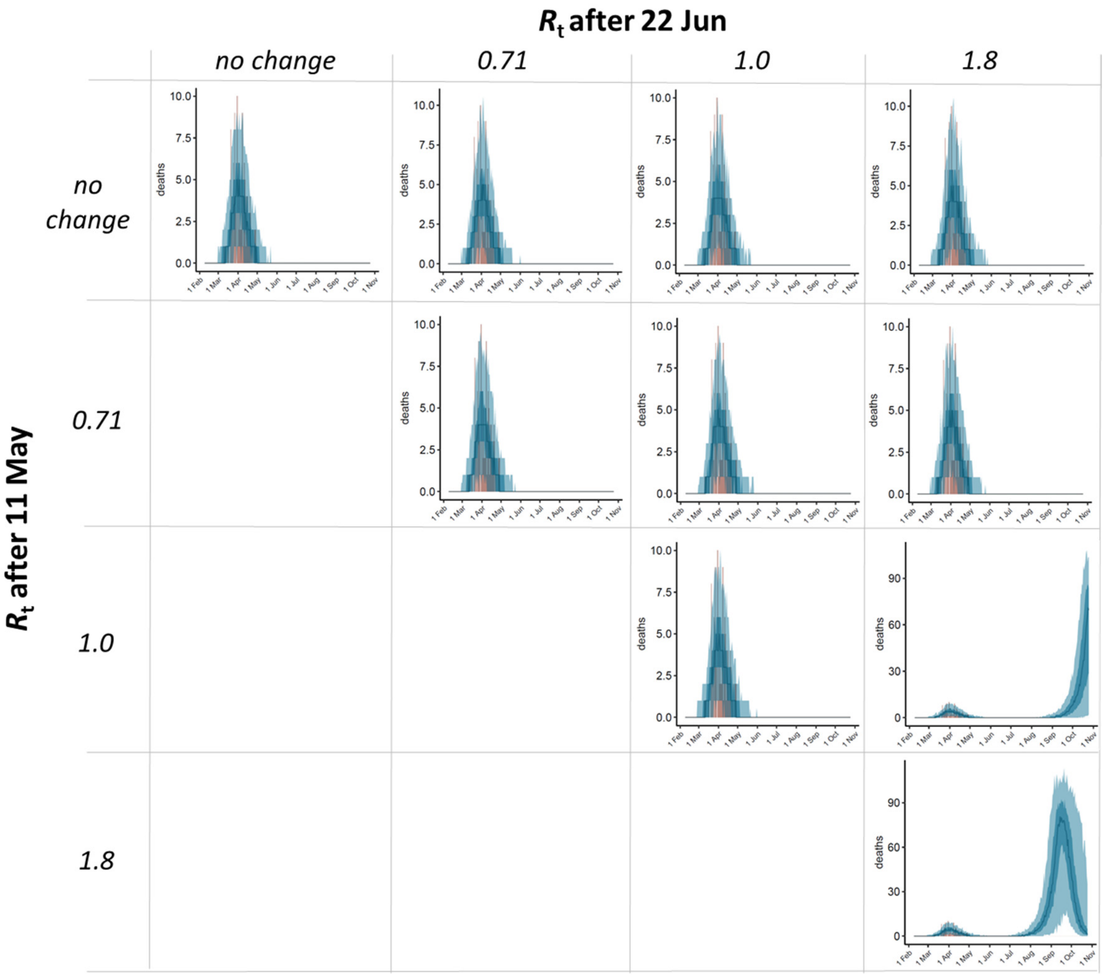

Figure S4. Forecast for daily deaths in La Rioja (excluding data from elderly retirement homes with reported cases) over the upcoming months in different scenarios. Blue bands (light blue 95% CI; dark blue 50% CI) represent the estimated daily deaths over time, computed as 100 discrete random distributions from sets of parameters obtained by the model (R_0, R_t after main intervention, and initial number of infections; Table 3), for different scenarios depending on possible changes in R_t after the proposed modifications in the intervention measures in two key dates (11 May and 22 June). Reported deaths to date are represented as brown bars.

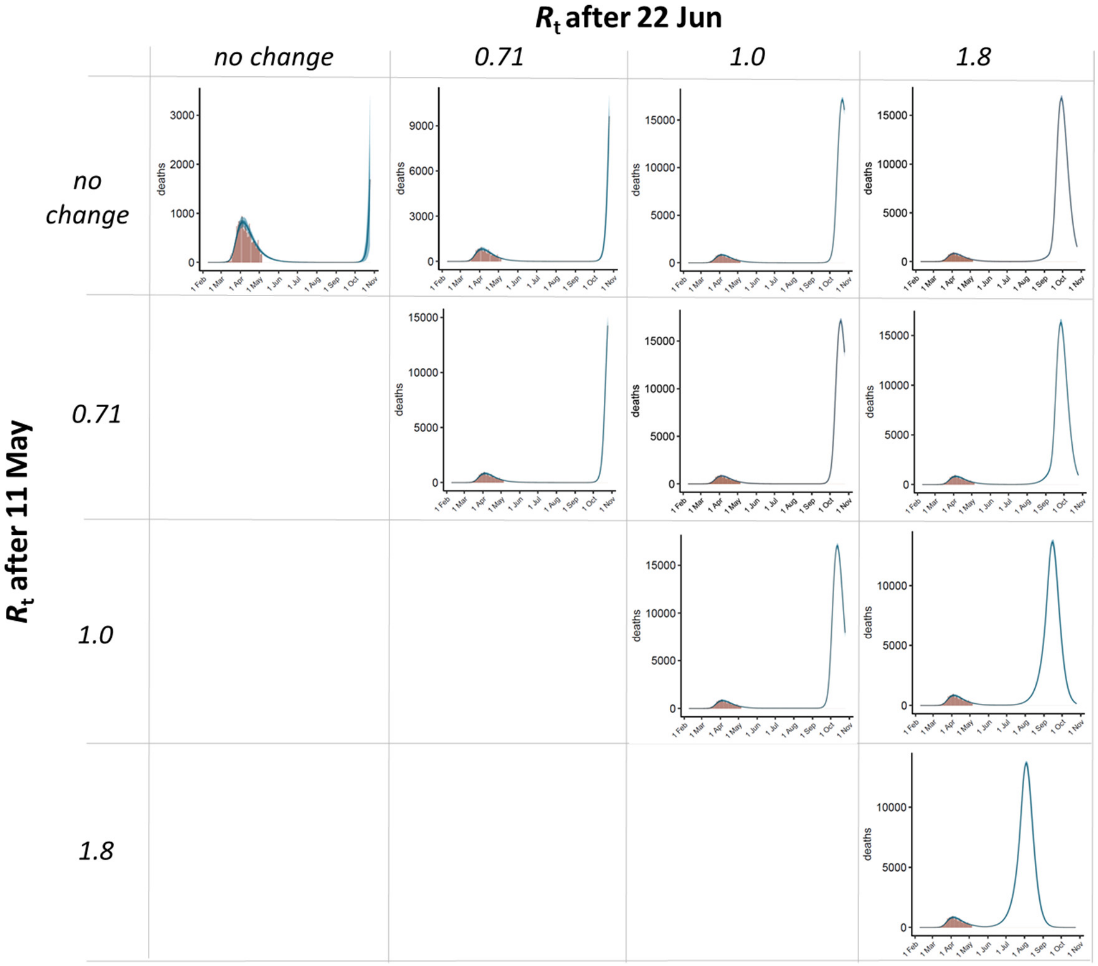

Figure S5. Forecast for daily deaths in Spain over the upcoming months in different scenarios, assuming full return to pre-pandemic period on 1 September 2020. Blue bands (light blue 95% CI; dark blue 50% CI) represent the estimated daily deaths over time, computed as 100 discrete random distributions from sets of parameters obtained by the model (R_0, R_t after main intervention, and initial number of infections; Table 3), for different scenarios depending on possible changes in R_t after the proposed modifications in the intervention measures in two key dates (11 May and 22 June). Reported deaths to date are represented as brown bars.

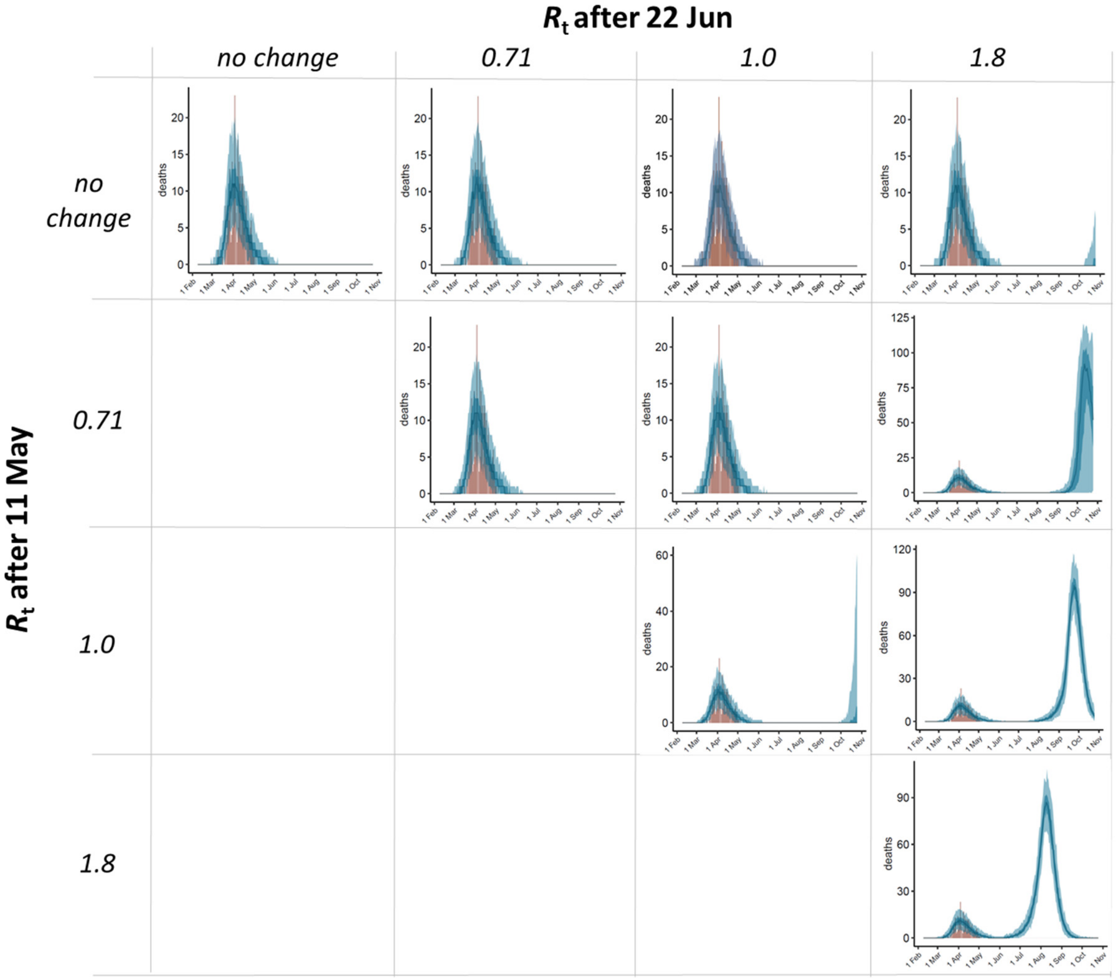

Figure S6. Forecast for daily deaths in La Rioja over the upcoming months in different scenarios, assuming full return to pre-pandemic period on 1 September 2020. Blue bands (light blue 95% CI; dark blue 50% CI) represent the estimated daily deaths over time, computed as 100 discrete random distributions from sets of parameters obtained by the model (R_0, R_t after main intervention, and initial number of infections; Table 3), for different scenarios depending on possible changes in R_t after the proposed modifications in the intervention measures in two key dates (11 May and 22 June). Reported deaths to date are represented as brown bars.

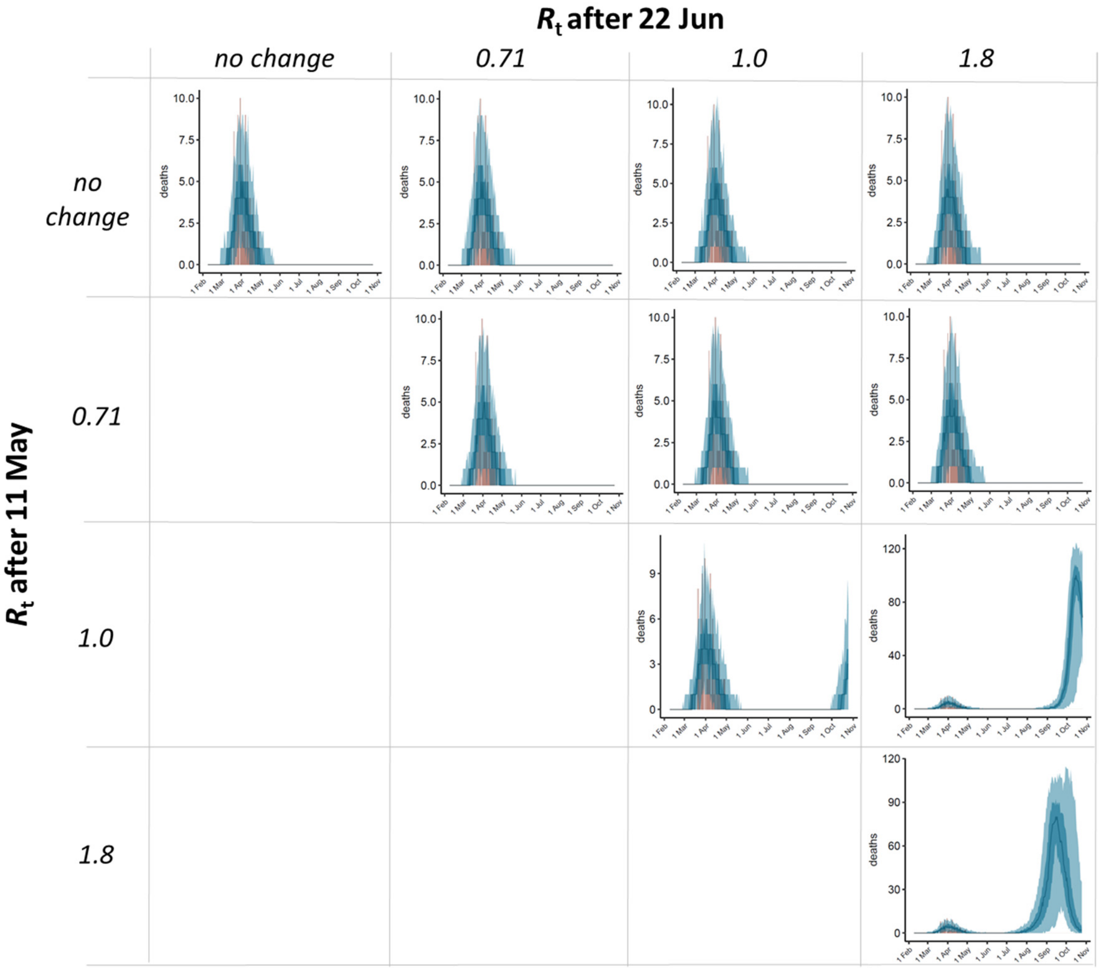

Figure S7. Forecast for daily deaths in La Rioja (after excluding data from elderly retirement homes with reported cases) over the upcoming months in different scenarios, assuming full return to pre-pandemic period on 1 September 2020. Blue bands (light blue 95% CI; dark blue 50% CI) represent the estimated daily deaths over time, computed as 100 discrete random distributions from sets of parameters obtained by the model (R_0, R_t after main intervention, and initial number of infections; Table 3), for different scenarios depending on possible changes in R_t after the proposed modifications in the intervention measures in two key dates (11 May and 22 June). Reported deaths to date are represented as brown bars.
